# Impact of Cheese Micronutrient Fortification on Micronutrient Consumption in Children from Morocco: A Modelling Study

**DOI:** 10.3390/nu18091397

**Published:** 2026-04-29

**Authors:** Alba M. Santaliestra-Pasías, Isabel Rueda-De Torre, Mª Isabel Benedicto-Toboso, Luis Mariano Esteban, Sergio Sabroso-Lasa, Karima Sabounji, Larbi Rjimati, Luis A. Moreno

**Affiliations:** 1 Growth, Exercise, Nutrition and Development (GENUD-B34_23R) Research Group, Facultad de Ciencias de la Salud, Universidad de Zaragoza, C/Pedro Cerbuna 12, 50009 Zaragoza, Spainibenedicto@unizar.es (M.I.B.-T.); lmoreno@unizar.es (L.A.M.); 2 Instituto de Investigación Sanitaria de Aragón (IIS Aragón), 50009 Zaragoza, Spain; 3 Centro de Investigación Biomédica en Red de Fisiopatología de la Obesidad y Nutrición (CIBERObn), Instituto de Salud Carlos III, 28029 Madrid, Spain; 4 Instituto Agroalimentario de Aragón (IA2), Universidad de Zaragoza-CITA, 50013 Zaragoza, Spain; 5 Facultad de Ciencias de la Salud y del Deporte, Universidad de Zaragoza, 22001 Huesca, Spain; 6 Escuela Politécnica de La Almunia, Universidad de Zaragoza, 50100 Zaragoza, Spain; lmeste@unizar.es; 7 Biocomputation and Physics of Complex Systems, Universidad de Zaragoza, 50018 Zaragoza, Spain; 8 Genetic and Molecular Epidemiology Group (GMEG), Spanish National Cancer Research Centre (CNIO), 28029 Madrid, Spain; 9 International University of Casablanca, Casablanca 50169, Morocco

**Keywords:** food fortification, micronutrient intake, children, dairy products, processed cheese, dietary modelling

## Abstract

**Background/Objectives**: Micronutrient malnutrition, particularly deficiencies in calcium, vitamin D, iron, zinc, and iodine, remains a significant public health issue among school-aged children in Morocco. Processed cheese, such as “The Laughing Cow” (TLC), has potential as a vehicle for fortification due to its widespread consumption and accessibility. This study aimed to evaluate the impact of fortified TLC on micronutrient intake and adequacy relative to the Recommended Dietary Allowances (RDA), among Moroccan children aged 6–12 years, and to explore differences in effects by socioeconomic status (SES). **Methods**: Data from the Moroccan Household Budget Survey (2013–2014) included 9266 children (39.4% TLC consumers). Dietary intake was assessed using 24 h recalls, and nutrient composition was analyzed using Ciqual 2020 tables and specialized software. Fortification scenarios were modelled to estimate potential impacts on micronutrient intake and compliance with RDAs. **Results**: Under the modelling scenarios, consumption of one portion/day of fortified TLC significantly improved RDAs compliance for iron, iodine, and zinc (*p* < 0.05). There was also an increase in RDA compliance for calcium and vitamin D, but differences were not significant. The impact of fortification on micronutrient intake and RDA compliance increased with socioeconomic status. Consumers of more than one portion/day showed the highest compliance with RDAs (*p* < 0.001). Fortification effects were consistent across age subgroups. **Conclusions**: Fortifying processed cheese represents a feasible strategy to address micronutrient deficiencies among Moroccan schoolchildren. This study highlights the potential of targeted fortification programmes to improve public health outcomes, particularly in vulnerable populations. Further research is needed to optimize fortification approaches and ensure sustainability.

## 1. Introduction

Since the early 2000s, the World Health Report has identified deficiencies in key micronutrients such as iodine, iron, vitamin A, and zinc as some of the leading global health risk factors [1]. These deficiencies, collectively referred to as micronutrient malnutrition, have significantly contributed to the global burden of disease, particularly in low- and middle-income countries [2]. Their prevalence is especially pronounced among vulnerable populations, including children, pregnant women, and individuals living in food-insecure regions. Over the past decades, efforts to address these deficiencies have become a cornerstone of global health and nutrition policies.

Micronutrient malnutrition poses substantial challenges due to its widespread impact on physical and cognitive development, immune function, and overall health outcomes. To combat these issues, two primary strategies have been identified: micronutrient enrichment and fortification. Although the terms “enrichment” and “fortification” are often used interchangeably, subtle differences exist. Enrichment typically refers to the addition of micronutrients to food products, whether or not these nutrients were present in the original food prior to processing. On the other hand, fortification is a deliberate practice aimed at increasing the levels of essential micronutrients—such as vitamins and minerals—in a food product to improve its nutritional quality. This approach has been recognized for its potential to provide public health benefits with minimal risks, particularly when implemented as part of comprehensive nutrition programmes targeting at-risk populations [3,4,5].

Food fortification has long been recognized as a strategic approach to address micronutrient malnutrition on a population level [6,7,8,9,10]. However, the effectiveness of fortification initiatives is limited when significant portions of the target population—whether due to poverty, geographic isolation, or lack of awareness—have minimal or no access to fortified foods. A comprehensive and context-specific planning of food fortification programmes is crucial to ensure their success [11]. This requires assessing both the potential nutritional impact on the population and the feasibility of program implementation. The success of such programmes is measured not only by their public health outcomes, such as reductions in micronutrient deficiencies, but also by their sustainability over time. This sustainability depends on multi-sectoral collaboration, involving national public health authorities, researchers, policymakers, the private sector, educators, trade and legal entities, non-governmental organizations, and commercial industries. The active involvement of the private sector, food manufacturers, and civil society is particularly essential in driving innovation, ensuring accessibility, and achieving wide-scale adoption of fortified products.

The national nutrition survey conducted by the Ministry of Health in Morocco in 2019/2020 showed that 2.9% of children aged 6 to 12 experienced wasting, 11.3% suffered from stunting, and 18.8% had overweight, including 5.5% with obesity [12]. The Dietary Diversity Score (DDS) was lower in rural areas, where starchy foods, fruits, and vegetables were predominant, while milk and dairy consumption was more frequent in urban areas. The DDS is an indicator of diet quality based on the number of different food groups consumed over a 24 h period. It is calculated by summing the food groups reported, with higher scores indicating greater diversity and potentially nutritional adequacy [13]. According to the World Health Organization (WHO) classification, the national prevalence of anemia is 23.8%, with 14.2% of cases being moderate to severe [12], which is higher in urban areas (16.9%) than rural areas (10.9%). Iron deficiency affects 11.9% of children, and 23.7% of children with anemia. Additionally, 3.1% of children had vitamin D deficiency, with 27.3% showing insufficiency. Moreover, this survey showed that iodine deficiency in children is within the limit range of UNICEF recommendations [14]. Indeed, creatinine-corrected iodide showed moderate and severe iodine deficiency (<50 µg/g Creatinine) in 21.6% of children in this age group. Vitamin A deficiency affects 10.9% of children, with higher proportions in rural areas (12.9%), compared to urban areas (9%).

The primary dietary objective of food fortification is to ensure that the majority of individuals within a high-risk population group (approximately 97.5%) receive adequate levels of specific micronutrients without exceeding safe intake levels for the population as a whole. In Morocco, cheese has been identified as a promising vehicle for fortification due to its widespread consumption and accessibility. In 2018, the per capita consumption of all types of cheese in Morocco was estimated at 1.6 kg per year, with approximately 81% of Moroccans consuming processed cheese [15]. Urban populations showed higher consumption rates (86%) compared to rural populations (75%). Among the various cheese products, spreadable processed cheese, such as TLC, dominates consumer preferences, accounting for 75% of total cheese consumption [12]. These products are widely accessible across social classes and are marketed to families, including both adults and children over the age of three. With an average price of 1–1.2 Moroccan dirhams (approximately €0.09–€0.11) per portion, processed cheese is affordable for most households. Additionally, its long shelf life (up to six months) and ability to remain stable without refrigeration make it particularly suited for distribution in remote or underserved regions lacking cold storage facilities. The fortification of processed cheese provides an opportunity to enhance dietary intake of key micronutrients such as calcium, vitamin D, zinc, iron, and iodine, addressing nutritional gaps in an efficient and culturally acceptable manner. Despite the existence of modelling studies in other settings, evidence specific to the Moroccan context remains limited.

For all these reasons, the primary objective of this study is to evaluate the potential impact of systematic food fortification, particularly using “The Laughing Cow” (TLC) processed cheese fortified with calcium, vitamin D, iron, zinc, and iodine, on the dietary micronutrient intake of Moroccan schoolchildren aged 6 to 12 years. Specifically, the study builds upon existing modelling frameworks to: (1) Analyze the baseline consumption of key micronutrients (calcium, vitamin D, iron, zinc, iodine) among schoolchildren consuming non-fortified TLC; (2) Assess the impact of enriched TLC consumption on the overall micronutrient intake of this population, while examining potential differential effects by socioeconomic status; and (3) Provide information for the design and implementation of fortification programmes targeting vulnerable populations, contributing to the broader evidence supporting sustainable and effective public health interventions for micronutrient deficiencies.

## 2. Materials and Methods

### 2.1. Study Population

A Household Budget survey was performed in Morocco by the Household Surveys Division of the Statistics Directorate (High Planning Commission) between July 2013 and June 2014. The survey’s sampling plan was based on the general population and housing census of 2004. The sampling protocol followed the principles of a two-stage stratified survey. The sample selected for this survey comprises 15,970 households (10,380 Urban, 5590 Rural). Data collection was spread over 12 months, in 6 periods of 5 survey weeks of 12 days each. The sample for each period was representative of the different socio-economic categories and regions of the country. Average annual expenditure per person was used as a proxy-indicator of socioeconomic status. This variable was subdivided into quintiles (1 DH = 0.09 euros). The total sample size included in the study will be those children aged 6 to 12 years (n = 9266, 65.1% females). This study was conducted using a simulation-based modelling approach based on the micronutrient consumption in 2013–2014 (original formulation TLC) and the estimation of the potential impact of TLC fortification on that micronutrient consumption. No intervention period was conducted.

The National Survey on Household Consumption and Expenditure was conducted by the Haut Commissariat au Plan (Household Survey Division) under Visa Nº 28.03.13.01 and approved on 26 March 2012 by the Ethics Committee of the Faculty of Medicine and Pharmacy of the University of Rabat (Morocco). The population was informed about the possibility of participating in this survey. Participants gave their verbal consent before the start of the survey and were informed that they were free to participate or not and could withdraw at any time, without having to provide a reason.

### 2.2. Assessment of Food and Micronutrient Consumption

For each participant, 24 h dietary recalls were obtained during the week of the household visit. Trained health professionals performed the interviews. Information was obtained by direct interviews. Families were also advised to take notes about their consumption in the previous days, in order not to miss any food consumption information. Food portions were estimated using a photo album of foods usually consumed in Morocco and models of plates and glasses [16].

In order to assess nutrient intake, the French food composition table Ciqual 2020 [17] was used. It was selected due to the lack of a comprehensive national database in Morocco and its standardized data. Its broad coverage of essential micronutrients makes it appropriate for the Moroccan context. Foods usually consumed in Morocco were categorized according to the Ciqual 2020 classifications by expert Nutritionists. The final nutrient composition analysis was directly performed by the Nutrilog society using their own software (Marans, France) [18].

### 2.3. Composition of Spreadable Cheese

The composition of spreadable cheese at the time of the surveys was considered for the initial nutrient composition analysis. The initial nutritional information of spreadable cheese (TLC) corresponded to 2013. The fortified nutritional information used for the modelling study and the prediction of the consumption was based on the nutritional composition and fortification of 2020. The modelling study has been developed with the current nutritional composition (Table 1).

### 2.4. Statistical Analysis

The nutrient composition of fortified cheese (2020) has been considered, as compared with the original formulation (2013), making a specific effort on the vitamins and minerals fortified. Macro- and micronutrient compositions were calculated taking into consideration both scenarios (2013, original, and 2020, fortified), without changing the food quantities reported as consumed.

Mean daily intakes of calcium, vitamin D, iron, zinc and iodine derived from the 24 h dietary recalls were compared between original intakes and the fortification scenario. Also, compliance with national dietary recommendations was examined based on WHO guidelines [19]. Differences in mean micronutrient intakes between the original and the fortification scenario were tested through the use of paired *t*-tests or the Mann–Whitney-Wilcoxon test. Also, the Kruskall–Wallis test was used to compare groups. Differences in the proportions of the populations meeting the dietary recommendations were tested with the McNemar test. Linear regression models were applied to predict cheese intake complying with the calcium, vitamin D, iron, zinc, and iodine RDA.

To estimate the consumption needed to meet the requirements, a linear adjustment in solid line was performed, estimated by linear regression models, using the consumption as the predictor variable and the features Energy, Calcium, Iron, Iodide, Zinc and Vitamin D as the variables to predict in each case. The linear model provides an average of the behaviour of all cases, so that for a value on the y-axis of the requirement, the value on the x-axis of necessary cheese consumption to meet the requirement can be estimated. Additionally, a more accurate nonlinear adjustment in a dashed line was estimated by LOESS (locally estimated scatterplot smoothing). At each point in the range of the data set, a low-degree polynomial is fitted to a subset of the data, with explanatory variable values near the point whose response is being estimated. The polynomial is fitted, giving more weight to points near the point whose response is being estimated and less weight to points further away. The value of the regression function for the point is then obtained by evaluating the local polynomial using the explanatory variable values for that data point. The LOESS fit is complete after regression function values have been computed for each of the n data points. This adjustment provides a more accurate performance, but with very similar values for the requirement needed. Thus, for simpler interpretability, the linear model was used to estimate cheese consumption requirements.

## 3. Results

Among the 9266 participants aged 6 to 12 years, 3652 (39.4%) reported consuming some quantity of spreadable cheese. Table 2 presents the total daily energy and micronutrient intake for two scenarios: consumption of the original formulation of TLC versus fortified cheese formulation. These values represent the participant’s overall dietary intake, with the respective cheese formulation integrated into their total diet. No significant differences in overall nutritional intake were observed between these two scenarios.

Table 3 outlines the proportion of participants meeting the current Recommended Dietary Allowances (RDA) under both consumption scenarios (original and fortified cheese). Overall, no differences were identified in RDA compliance between these groups for any of the micronutrients evaluated. For iron and iodine, a slight improvement in compliance was noted under the fortified scenario, though the changes did not reach statistical significance.

**Figure 1:**
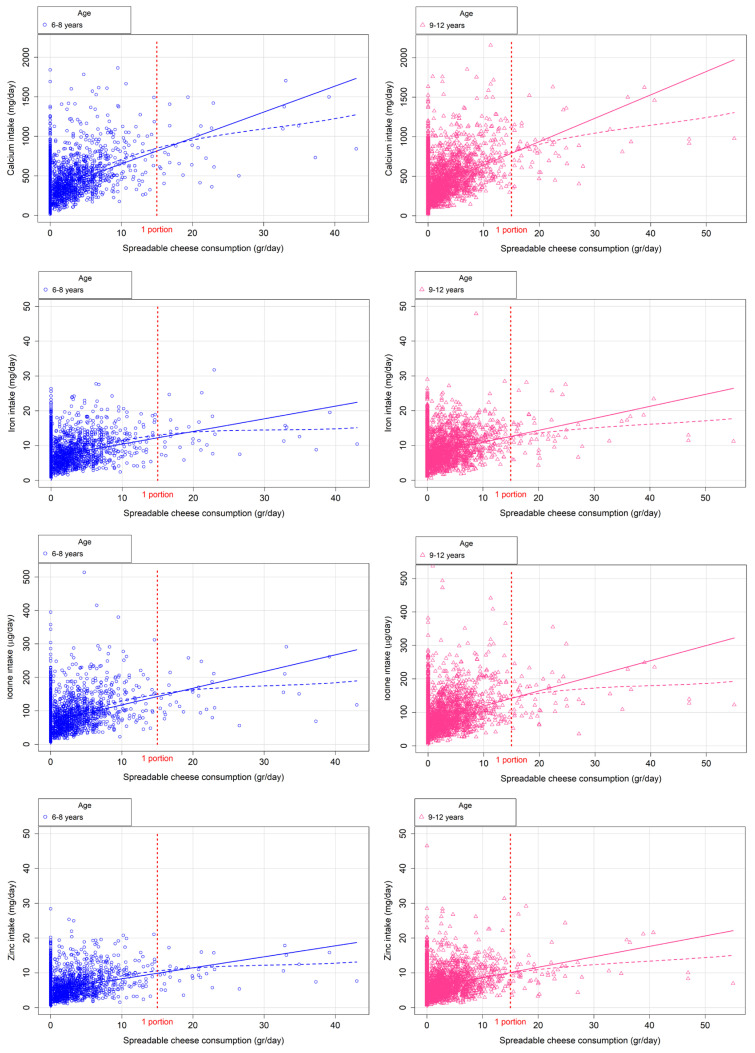
Fortified spreadable cheese consumption to achieve calcium, iron, iodine and zinc RDAs by age group. These figures should be interpreted as theoretical estimations of the amount of fortified cheese required to reach RDA thresholds, rather than observed consumption patterns. The solid line represents the linear regression model, while the dashed line corresponds to the LOESS smoothing. RDA: Recommended Dietary Allowance.

**Figure 2:**
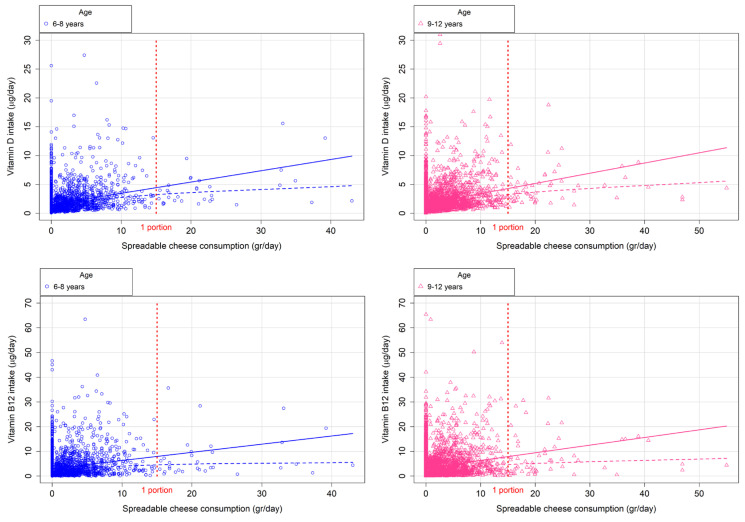
Fortified spreadable cheese consumption to achieve the vitamin D and vitamin B12 RDAs, by age group. These figures should be interpreted as theoretical estimations of the amount of fortified cheese required to reach RDA thresholds, rather than observed consumption patterns. The solid line represents the linear regression model, while the dashed line corresponds to the LOESS smoothing. RDA: Recommended Dietary Allowance.

Figure 1 and Figure 2 illustrate the extent to which consumers of fortified spreadable cheese meet RDAs for key minerals (e.g., calcium, iron, iodine, and zinc) and vitamins (e.g., vitamin D and vitamin B12), stratified by age group (6–8 years and 9–12 years). Each figure highlights the potential contribution of one portion of fortified spreadable cheese to increasing RDA compliance.

Table 4 details the proportion of participants achieving RDA compliance under two modelling scenarios. In the first, the original spreadable cheese reported in the dietary recalls was replaced by an equivalent amount of fortified cheese. In the second, a daily 15 g of fortified spreadable cheese was added to the reported diet. The inclusion of one portion per day significantly increased the proportion of children meeting RDAs for iron, iodine, and zinc (*p* < 0.05). Despite an increase in compliance with RDAs for calcium and vitamin D, no significant differences were observed. Table 5 provides an analysis of the estimated daily intake of fortified spreadable cheese (in grams) required by each age group to achieve RDA compliance for each studied micronutrient. For most nutrients, the estimated requirements fall within or near the standard portion size of 15 g/day. In contrast, the intake required to meet the RDA for vitamin D substantially exceeds one portion in both groups.

**Figure 3:**
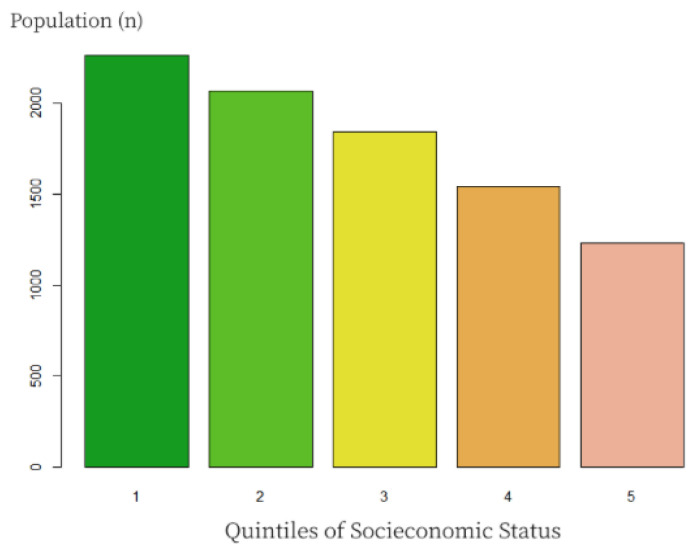
Distribution of the studied population by socioeconomic status (income). National Quintiles in 2014 (1 DH = 0.09 euros). Q1: <7149.77 DH; Q2: 7149.77 to 9964.29 DH; Q3: 9964.3 to 13,639.8 DH; Q4: 13,639.8 to 20,395 DH; Q5: >20,395 DH.

Analyzing the distribution of the studied population by socioeconomic status, Figure 3 shows the proportions by socioeconomic status quintiles. The highest proportion of the population was included in the three lowest quintiles. The total daily micronutrient intake and the proportion of children complying with the current RDA by quintiles of socioeconomic status, considering the consumption of fortified spreadable cheese (2020), are displayed in Table 6. An increasing gradient of micronutrient consumption and RDA compliance was observed by quintiles of socioeconomic status (*p* < 0.05). An additional comparison was developed to compare the compliance with micronutrient RDAs between consumers and non-consumers of fortified spreadable cheese, and to compare consumption of less than one portion per day or more (Table 7). The results showed statistically significant differences between groups in terms of compliance with current RDAs, with a higher compliance among those who consume spreadable cheese and those who consume more than one portion of cheese per day (*p* < 0.001).

## 4. Discussion

The modelling study showed that cheese fortification in Morocco may have an impact on micronutrient consumption in school-age children. Available studies, including 20 out of 43 conducted in low- and middle-income countries, showed that food fortification with multiple micronutrients had low to very low-quality evidence for health outcomes improvements in men, women, and children [20]. However, large-scale food fortification with key micronutrients may improve micronutrient and health status in women and children in low- and middle-income countries [21]. In this sense, fortifying dairy products with micronutrients is an approach designed to decrease nutritional deficiencies, especially among vulnerable population groups like children and adolescents. These products may have a positive impact on children’s health by addressing micronutrient deficiencies and enhancing growth indicators.

In Morocco, food fortification has been identified as a key strategy to address micronutrient deficiencies. According to the National Nutrition Action Plan (PNN), by 2030, the country aims to achieve the following: (1) reduce iron deficiency by one-third compared to 2000 levels, (2) decrease zinc deficiency, (3) eliminate iodine deficiency disorders, and (4) reduce vitamin D deficiency [22].

Milk and cheese, as widely consumed foods with beneficial nutritional properties, provide an effective means of delivering key vitamins and minerals. However, the degree of these benefits depends on the specific nutrients included and the characteristics of the target population. Fortification of cheese entails the incorporation of micronutrients, either directly or through probiotics, to enhance its nutritional and technological properties. Additionally, the use of probiotics and agri-food industry by-products contributes to both eco-sustainability and consumer well-being [23].

Based on data from the Moroccan consumption survey conducted in 2013–2014, a significant proportion of children (60.6%) do not consume spreadable cheese. However, the introduction of fortified spreadable cheese has been shown to increase micronutrient intake. No significant differences were observed in total dietary intake or in the proportion of children meeting the RDAs. Consumers of spreadable cheese showed higher compliance with RDAs for all analyzed micronutrients, compared to non-consumers. The current modelling analyses suggest that regular consumption of fortified spreadable cheese could substantially contribute to achieving RDA compliance for essential micronutrients. The inclusion of a 15 g portion of fortified spreadable cheese in the daily diet of children could significantly enhance RDA compliance, especially for key nutrients such as iron, iodine, zinc, and vitamin D. However, it is important to note that, while the estimated intake required to meet RDAs is within realistic consumption ranges for most micronutrients, substantially higher amounts are needed for vitamin D. Its long shelf life (up to six months) and stability at room temperature makes it an ideal product for distribution in remote or underserved regions lacking refrigeration infrastructure, addressing logistical barriers to improving nutritional outcomes in these populations. However, it is important to consider that improvements in iron intake and RDA compliance do not necessarily translate into proportional improvements in physiological iron status. The bioavailability of dietary iron may be influenced by other components of the diet, particularly calcium, which is present in dairy products and may inhibit the absorption of both heme and non-heme iron [24]. In this context, the absorption of non-heme iron is strongly affected by dietary factors such as vitamin C, which can enhance its bioavailability [25]. Therefore, adequate consumption of fruits and vegetables rich in vitamin C may represent a complementary strategy to improve iron status and reduce the risk of iron deficiency anemia. Micronutrient intake and compliance with recommendations were found to be lower among children from low-income households. Fortified spreadable cheese represents an affordable and accessible solution to improve RDA compliance, particularly in vulnerable populations.

A systematic review evaluated the effects of micronutrient-fortified dairy products on the health of children and adolescents [26]. The findings indicated a slight, non-significant improvement in hemoglobin levels and a reduction in iron deficiency anemia; however, the overall health benefits were minimal, and evidence on cognitive or functional outcomes was of very low quality. Another systematic review focused on infants and young children concluded that multi-micronutrient fortification of milk and cereal products effectively reduced anemia and improved hemoglobin levels [27], although evidence for other health outcomes remained inconclusive.

A randomized controlled trial revealed that fortified milk (including vitamin A, E, C, iron, zinc, selenium, and copper) notably enhanced growth and iron status in children aged 1 to 4 years [28]. Another study in Nigerian toddlers found that fortified dairy-based products improved in vitamin A, D, and selenium status, particularly at higher intake levels [29].

These findings partially support the rationale behind our modelling study and highlight the need for longer intervention periods to detect significant impacts on growth and cognitive development. Although similar modelling studies have been conducted in other settings, the relevance of this study lies in its context-specific application to Morocco, where dietary patterns, food accessibility, and socioeconomic disparities may influence both the feasibility and the potential impact of fortification strategies.

A recent systematic review with meta-analysis also shows that vitamin A supplementation is thought to improve mortality largely by decreasing childhood infections; iron supplementation interventions improved all indicators of iron status (hemoglobin concentration, serum/plasma ferritin, anemia, iron deficiency, and iron-deficiency anemia); zinc supplementation work to reduce diarrhea; and early nutrition and fortification positively impact mental and motor development outcomes [30].

Fortified milk consumption has shown effectiveness in lowering anemia risk by increasing hemoglobin and ferritin concentrations. This impact is particularly notable in low-income regions where deficiencies are widespread. Also, taking into consideration fortified dairy products support weight and height increases in children, particularly among those in low-income settings or with extended consumption durations [28,31,32]. They serve as a reliable and effective source of complementary nutrition, fostering healthy growth trajectories [32,33]. Fortified milk consumption has been linked to moderate improvements in weight and height gain among young children, particularly with prolonged intake. Fortified milk enriched with micronutrients and omega-3 fatty acids has been shown to improve cognitive functions, including working memory and processing speed, in children [34].

Fortified dairy products act as an effective dietary supplement, enhancing overall nutrient intake, particularly in areas where children face deficiencies due to inadequate diets [31,35]. In the case of milk, it has been shown to lower the prevalence of common illnesses, such as diarrhea and respiratory infections, particularly in younger children, indicating its potential to enhance overall health and reduce morbidity. Moreover, this kind of product provides a sustainable solution for improving children’s nutritional status, serving as a practical alternative to direct supplementation initiatives [28].

Fortifying dairy products, such as milk and cheese, presents a valuable strategy for combating nutritional deficiencies. Although certain studies report benefits in reducing anemia and supporting growth, the broader health effects, particularly on cognitive and functional outcomes, remain underexplored. To optimize their impact, fortification efforts should be customized to the needs of specific populations and settings. For instance, it plays a crucial role in enhancing levels of key micronutrients, including iron, zinc, vitamin A, and vitamin D, which are commonly lacking in children, and helps align children’s dietary intake with recommended levels [29,35].

Home fortification may also be used as an effective method to improve hemoglobin, iron, and zinc status. Although this new approach improves a broader range of micronutrients as well as child growth indices, it is essential to coordinate both individual and public health strategies to ensure the acquisition of adequate micronutrient levels, especially at early ages. Policy makers’ sensitivity and commitment to the nutritional health of the population can be considered a good sign and investment for health promotion and prevention of malnutrition and chronic diseases later in life. In this sense, food fortification could increase specific nutrient intake, owing to claims for its relative ease and non-obtrusive nature, which requires no behavioural change on the part of the beneficiary. This study presents several limitations. First, the dietary data were collected in 2013–2014 and may not fully reflect current consumption patterns. Second, nutrient intake was estimated using the French Ciqual 2020 composition database, which, although comprehensive and standardized, may not entirely capture the specific nutritional profile of food consumed in Morocco. Additionally, dietary intake was based on a single-hour recall, which may not reflect usual intake due to intra-individual variability. However, as the same data were applied across both scenarios, it is not expected to introduce a significant bias.

## 5. Conclusions

This modelling study suggests that the inclusion of fortified dairy products, such as the fortified spreadable cheese evaluated in this study, may represent a promising strategy to support micronutrient intake in children. These products offer a practical and potentially sustainable approach to enhancing children’s nutritional health, especially among vulnerable groups. While their effects on cognitive development and other health outcomes remain uncertain, potential benefits may be more pronounced in low-income settings and with consistent long-term consumption. From a health promotion perspective, the inclusion of one portion of fortified spreadable cheese may contribute to improving micronutrient status without exceeding nutritional requirements.

## Figures and Tables

**Table 1:** Nutritional composition of the “The Laughing Cow” (TLC) spreadable cheese per 100 g and per 15 g.

	per 100 g		per 15 g	
Macro- and micro nutrient composition	2013	2020	2013	2020
Energy (KJ)	1120	890	168	134
Energy (Kcal)	271	215	40.7	32.3
Proteins (g)	10	9	1.5	1.4
Fats (g)	23	17	3.5	2.6
Saturated fats (g)	15.5	11.5	2.3	1.7
Carbohydrates (g)	6	6.5	0.9	1.0
Sugars (g)	6	6.5	0.9	1.0
Salt (g)	1.7	1.7	0.3	0.3
Calcium (mg)	630 (60% RDA)	700 (87.5% RDA)	94.5 (9% RDA)	105 (13.1% RDA)
Iron (mg)	-	4.2 (30% RDA)	-	0.6 (4.5% RDA)
Iodine (µg)	-	22.5 (15% RDA)	-	3.4 (2.3% RDA)
Zinc (mg)	0.8 (15% RDA)	2.3 (22% RDA)	0.12 (2.3% RDA)	0.3 (3.3% RDA)
Vitamin D (µg)	1.3 (15% RDA)	2.5 (66% RDA)	0.2 (2.3% RDA)	0.4 (9.9% RDA)
Vitamin B12 (µg)	0.5 (21% RDA)	-	0.1 (3.2% RDA)	-

Standard portion size = 15 g. RDA: Recommended Dietary Allowance. Statistical significance was set at *p* < 0.05.

**Table 2:** Total daily energy and micronutrient intake by spreadable cheese composition (original or fortified).

Median, IQR	Original	Fortified	*p*-Value
Energy (kcal)	1536.0 (1095.0–2083.0)	1533.6 (1092.2–2076.7)	0.799
Calcium (mg)	328.0 (221.0–481.0)	329.0 (222.0–483.0)	0.737
Iron (mg)	6.8 (4.8–9.7)	6.9 (4.8–9.8)	0.362
Iodine (μg)	67.7 (45.6–98.4)	67.9 (45.8–98.8)	0.662
Zinc (mg)	5.1 (3.5–7.4)	5.1 (3.5–7.4)	0.959
Vitamin D (μg)	1.2 (0.6–2.2)	1.3 (0.7–2.3)	0.082
Vitamin B12 (μg)	2.1 (0.8–4.4)	2.1 (0.8–4.4)	0.850

The Mann–Whitney–Wilcoxon test has been used to check if there are significant differences between the two scenarios. Statistical significance was set at *p* < 0.05.

**Table 3:** Proportion of children complying with the current Recommended Dietary Allowances (RDA).

	Original n (%)	Fortified n (%)	*p*-Value
Calcium	119 (1.3)	123 (1.4)	0.846
Iron	2883 (32.2)	2927 (32.7)	0.492
Iodine	1911 (21.4)	1929 (21.6)	0.757
Zinc	2951 (33.0)	2949 (33.0)	0.987
Vitamin D	26 (0.3)	27 (0.3)	0.999
Vitamin B12	5450 (60.9)	5436 (60.8)	0.842

Groups were compared using the McNemar test. RDA: Recommended Dietary Allowance. Statistical significance was set at *p* < 0.05.

**Table 4:** Proportion of participants meeting micronutrient RDAs under the fortified cheese scenario and with an additional 15 g/day portion.

	Fortified n (%)	Adding 15 g of Cheese Consumption n (%)	*p*-Value
Calcium	123 (1.4)	149 (1.7)	0.127
Iron	2927 (32.7)	3366 (37.6)	<0.001
Iodide	1929 (21.6)	2060 (23.0)	0.020
Zinc	2949 (33.0)	3261 (36.4)	<0.001
Vitamin D	27 (0.3)	29 (0.3)	0.894
Vitamin B12	5437 (60.8)	5437 (60.8)	-

The fortified scenario represents a substitution in which the original spreadable cheese reported in dietary recalls was replaced with an equivalent amount of fortified cheese. Groups were compared using the McNemar test. Statistical significance was set at *p* < 0.05.

**Table 5:** Estimated daily consumption of fortified spreadable cheese to meet the micronutrient RDAs, by age group.

Age Group	Calcium	Iron	Iodine	Zinc	Vitamin D
6–8 years	20.7 (19.4–21.9)	8.8 (8–9.6)	4.4 (4.1–4.8)	2.7 (2.4–3.0)	69.0 (62.6–75.4)
9–12 years	32.3 (30.6–33.9)	1.6 (1.3–1.9)	10.2 (9.5–10.9)	7.9 (7.3–8.5)	75.5 (69.0–81.9)

Data are expressed in grams (g) with 95% confidence intervals. RDA: Recommended Dietary Allowance. For reference, one portion = 15 g.

**Table 6:** Daily micronutrient intake and RDAs compliance by socioeconomic status quintiles, considering the fortified scenario (2020).

	Quintile 1		Quintile 2		Quintile 3		Quintile 4		Quintile 5		p */**
	$\bar{X}$ 95% C.I	n (%)	$\bar{X}$ 95% C.I	n (%)	$\bar{X}$ 95% C.I	n (%)	$\bar{X}$ 95% C.I	n (%)	$\bar{X}$ 95% C.I	n (%)	
Calcium (mg)	191 (137–255)	0 (0)	290 (222–378)	0 (0)	366 (280–478)	3 (0.2)	470 (352–599)	12 (0.8)	630 (457–840)	108 (8.8)	<0.001
Iron (mg)	4.48 (3.2–5.9)	135 (6.0)	6.5 (4.7–8.3)	483 (23.4)	7.6 (5.6–10.0)	673 (36.5)	9.15 (6.7–12.3)	819 (53.25)	10.71 (14.3–47.9)	816 (66.3)	<0.001
Iodine(μg)	39.8 (30.0–54.1)	22 (1.0)	61.6 (47.2–79.5)	169 (8.2)	75.6 (53.6–99.2)	383 (20.8)	95.1 (72.0–122.0)	609 (39.5)	122.5 (88.7–537.2)	746 (60.5)	<0.001
Zinc (mg)	3.2 (2.3–4.2)	125 (5.5)	4.7 (3.5–6.2)	448 (21.7)	5.6 (4.2–7.5)	683 (73.1)	6.9 (5.1–9.3)	832 (54.0)	8.6 (6.3–11.6)	861 (69.8)	<0.001
Vitamin D (μg)	0.6 (0.3–1.1)	0 (0)	1.1 (0.6–1.7)	2 (0.1)	1.4 (0.9–2.3)	1 (0.1)	1.9 (1.2–3.0)	4 (0.3)	5.1 (2.9–35.6)	20 (1.6)	<0.001
Vitamin B12 (μg)	0.8 (0.3–1.9)	765 (33.8)	1.8 (0.8–3.2)	1147 (55.5)	2.5 (1.1–4.6)	1237 (67.2)	3.4 (1.8–6.3)	1220 (79.2)	5.5 (2.5–11.0)	1067 (86.5)	<0.001

Micronutrient intake is expressed as the mean and 95% confidence interval. The proportion of children complying with the current Recommended Dietary Allowances (RDA) is shown as a number and percentage. * *p*-values estimated using the Kruskal–Wallis test to compare groups. ** Groups were compared using the McNemar test. Statistical significance was set at *p* < 0.05.

**Table 7:** Proportion of children meeting micronutrient RDAs, comparing fortified spreadable cheese consumers to non-consumers.

	Non-Consumers n (%)	Consumers n (%)	*p*-Value	Consumption Less Than 1 Portion * (<15 g) n (%)	Consumption More Than 1 Portion (15 g) n (%)	*p*-Value
Calcium	31 (0.5)	92 (3.3)	<0.001	104 (1.2)	19 (23.5)	<0.001
Iron	1681 (27.2)	1246 (45.2)	<0.001	2861 (32.3)	66 (81.5)	<0.001
Iodine	1003 (16.2)	926 (33.6)	<0.001	1869 (21.1)	60 (74.1)	<0.001
Zinc	1692 (27.3)	1257 (45.6)	<0.001	2879 (32.5)	70 (86.4)	<0.001
Vitamin D	9 (0.2)	18 (0.7)	<0.001	25 (0.3)	2 (2.5)	<0.001
Vitamin B12	3439 (55.6)	1997 (74.5)	<0.001	5365 (60.5)	71 (87.7)	<0.001

Groups were compared using the McNemar test. The table shows the proportion of children complying with the current micronutrients Recommended Dietary Allowance (RDA), comparing fortified spreadable cheese consumers to non-consumers, and * consumers of less than 1 portion/day (<15.6 g) to consumers of more than one portion/day (≥15.6 g). Statistical significance was set at *p* < 0.05.

## Data Availability

The data used in this study derive from the Moroccan Household Budget Survey (2013–2014) conducted by the Haut Commissariat au Plan and are not publicly available due to institutional and legal restrictions but may be available from the corresponding institutions upon reasonable request.

## Abbreviations

The following abbreviations are used in this manuscript:

CI	Confidence Interval
DDS	Diet Diversity Score
LOESS	Locally Estimated Scatterplot Smoothing
PNN	National Nutrition Action Plan
RDA	Recommended Dietary Allowances
SES	Socioeconomic Status
TLC	The Laughing Cow
WHO	World Health Organization
